# Late Renal Allograft Rupture Associated with Cessation of Immunosuppression following Graft Failure

**DOI:** 10.1155/2011/512893

**Published:** 2011-12-19

**Authors:** Sumayah Askandarani, Noura Aloudah, Hanan Al Enazi, Khaled O. Alsaad, Abdulrahman Altamimi

**Affiliations:** ^1^Division of Nephrology, Department of Medicine, King Abdulaziz Medical City, P.O. Box 22490, Riyadh 11426, Saudi Arabia; ^2^Department of Pathology and Laboratory Medicine, King Abdulaziz Medical City, P.O. Box 22490, Riyadh 11426, Saudi Arabia; ^3^King Abdullah International Medical Research Centre and College of Medicine, King Saud bin Abdulaziz University for Health Sciences, King Abdulaziz Medical City, P.O. Box 22490, Riyadh 11426, Saudi Arabia; ^4^Department of Hepatobiliary Sciences and Liver Transplantation, King Abdulaziz Medical City, P.O. Box 22490, Riyadh 11426, Saudi Arabia

## Abstract

A 29-year-old man developed chronic allograft nephropathy 63 months after renal transplantation. He became symptomatic with advanced chronic graft failure; his immunosuppressive medications were reduced and he was commenced on haemodialysis. Two months following the withdrawal of immunosuppression, he presented with abdominal pain, haematuria, and a marked drop in haemoglobin. The patient was taken to the operating room, where the renal allograft was found to be ruptured, and graft nephrectomy was subsequently performed. Histological examination of the graft specimen showed severe haemorrhagic acute vascular cellular rejection in a background of marked chronic allograft vasculopathy. Immunostaining for C4d showed diffuse, strong, linear circumferential staining of the peritubular capillaries, indicating a concurrent antibody-mediated rejection. We report herein an unusual case of spontaneous renal allograft rupture that occurred long time after transplantation due to severe acute rejection following cessation of immunosuppressive medications for advanced chronic allograft failure. To the best of our knowledge, the time interval between transplantation and the rupture of this allograft is the longest of those reported in the literature.

## 1. Introduction

Renal allograft rupture is a well-recognised but rare complication of renal transplantation, which normally occurs in the first few weeks after transplantation. It is associated with severe graft pain, hypotension, and a drop in haemoglobin. The most common cause of allograft rupture is acute rejection [1]. The incidence of allograft rupture has decreased due to the use of modern potent immunosuppressive medications [2–4]. Due to its devastating clinical course and outcome, recognition and prompt management of allograft rupture is important. Usually, nephrectomy is necessary treatment measure, but conservative surgical intervention has also been attempted to preserve the renal allograft in certain cases [2, 5–8]. We report an unusual case of late renal allograft rupture secondary to severe acute rejection, which followed cessation of the patient's immunosuppressive regimen due to advanced chronic allograft failure (CAF). To the best of our knowledge, the interval period of time between renal allograft transplantation and rupture in this case is the longest of those reported in the literature.

## 2. Case Report

A 29-year-old man received a living-unrelated kidney transplant abroad for end-stage kidney disease secondary to neurogenic bladder. The patient had an uneventful postoperative clinical course with good early graft function. The data of HLA matching and induction protocol are lacking. He was maintained on prednisone, cyclosporine, and myocophenolate mofetil. The patient was discharged on postoperative day 5, with an adequate urine output and serum creatinine 110 *μ*mol/L. In addition, he was kept on intermittent self-catherization four times per day. Forty-eight months after transplantation, the patient was diagnosed in another institution to have chronic allograft failure based on slow rising serum creatinine. No renal allograft biopsy was obtained *as the patient refused*. His immunosuppression was modified, whereby tacrolimus was substituted for cyclosporine A, when his serum creatinine had increased to 400 *μ*mol/L.

Sixty-three months after renal transplantation, the patient was first seen in our hospital, when he presented with hypoxic respiratory failure secondary to pneumonia, pulmonary oedema, and deteriorating kidney allograft function. His blood urea and nitrogen (BUN) was 44 mmol/L and serum creatinine 700 *μ*mol/L. He was admitted to the intensive care unit and started on ventilatory support. Appropriate antimicrobial coverage was started for pneumonia and haemodialysis was initiated. During the hospital admission for about a month, the patient was diagnosed to have advanced graft failure based on his clinical progression. The patient refused the renal allograft biopsy. He was maintained on haemodialysis and discharged on prednisone 5 mg daily. Tacrolimus and mycophenolate mofetil were discontinued.

**Figure 1:**
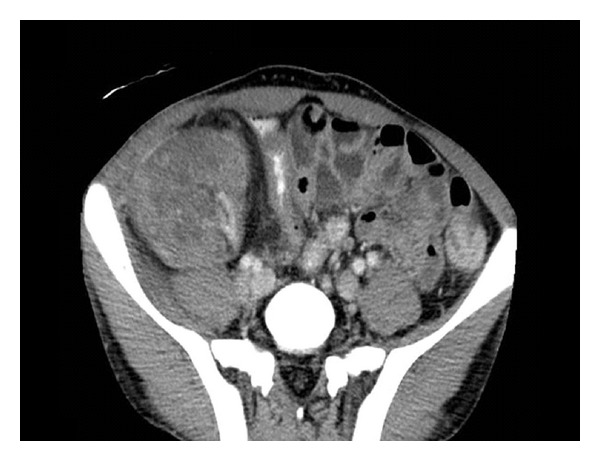
Computed tomography scan showing hyperdense areas in the renal allograft, in keeping with foci of parenchymal haemorrhage.

Two months later (65 months post renal transplant), the patient was admitted because of abdominal pain, graft tenderness, and gross haematuria. There was no history of abdominal trauma or recent renal allograft biopsy. Upon admission, the patient was pale, tachycardic, and hypotensive. His heart rate was 111 beat per minute, and the blood pressure 110/56 mmHg. Laboratory tests showed WBC of 2 × 103/*μ*L, haemoglobin of 5.5 g/dL (dropped from 12.7 g/dL one month earlier), haematocrit of 16.6%, platelets count of 344 × 103/Ul, PT of 9.1, PTT of 33.9, BUN of 13.0 mmol/L, and serum creatinine of 809 *μ*mol/L. The patient was resuscitated with crystalloid fluids and packed red blood cells. An enhanced abdominal computed tomography (CT) scan revealed few scattered hyperdense foci in the renal allograft, in keeping with microhaemorrhage and microperforations (Figure 1). The renal artery and vein were patent.

**Figure 2:**
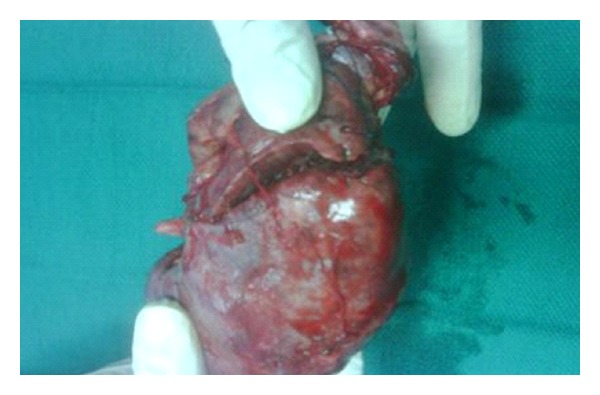
Oblique laceration on in the upper pole and mid portion of the kidney compatible with ruptured graft.

The patient was emergently taken to the operating room, where urinary bladder irrigation and washout revealed 3 litres of partially clotted blood. Exploration of the renal graft through the old transplant scar and intracapsular approach showed ruptured graft. An oblique laceration that measured 4 cm in length and involved the upper pole and the mid portion of the graft was evident (Figure 2). The renal artery and vein were dissected, clamped, and divided, along with the transplant ureter. The postoperative course was uneventful, and the patient was discharged 5 days after the graft nephrectomy.

**Figure 3:**
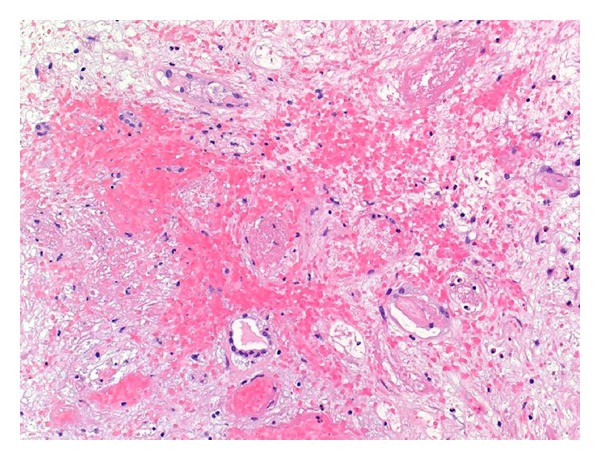
Marked interstitial oedema and haemorrhage (H&E ×100).

**Figure 4:**
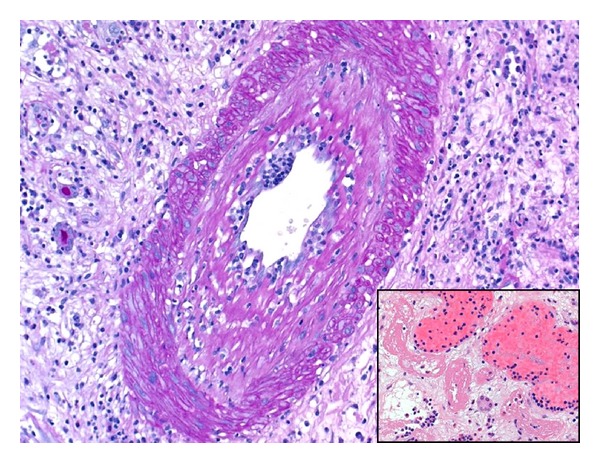
Severe endarteritis was readily identified in the interstitial blood vessels (PAS stain ×200), along with arterial and arteriolar fibrinoid necrosis (Inset H&E ×200).

**Figure 5:**
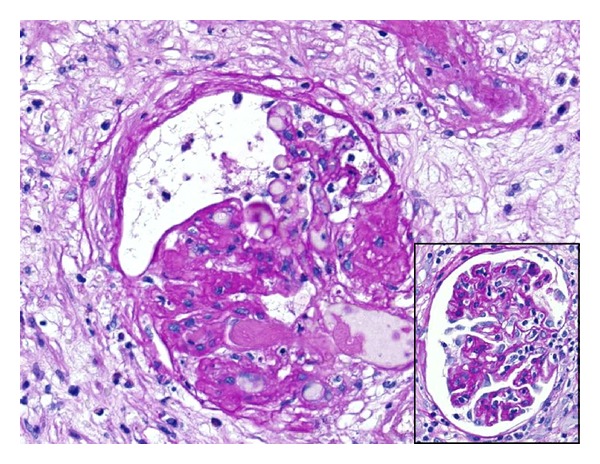
Fibrin microthrombi were also seen in the glomeruli (PAS ×200). Glomerulitis in the form of mononuclear inflammatory cells infiltrating the glomerular capillaries was seen (Inset PAS ×200).

**Figure 6:**
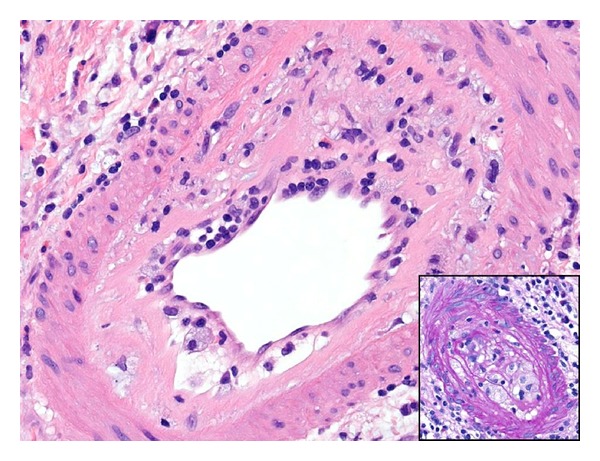
The morphological features of the acute rejection were found in a background of severe chronic allograft arteriopathy (H&E ×40, inset PAS ×200).

**Figure 7:**
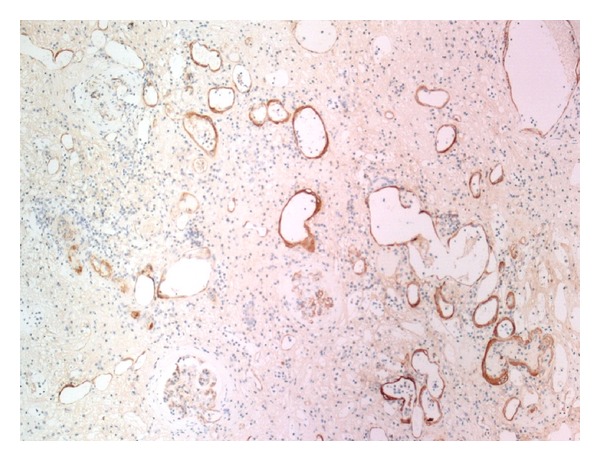
Immunohistochemical staining for C4d shows diffuse linear and strong circumferential staining in the peritubular capillaries, consistent with acute antibody-mediated rejection (C4d IHC ×100).

Histological examination showed morphological features compatible with severe acute vascular T-cell mediated (cellular) rejection, in a background of marked chronic allograft arteriopathy. The interstitium was markedly oedematous and showed areas of extensive haemorrhage (Figure 3). Severe interstitial inflammation was seen and the inflammatory infiltrate consisted of lymphocytes, plasma cells, and neutrophils. Large numbers of eosinophils were also noted. This was associated with severe *lymphocytic* tubulitis, acute tubular epithelial cell degenerative and regenerative changes in the viable renal tubules, and severe endarteritis with fibrinoid necrosis (Figure 4). The vast majority of glomeruli were globally sclerosed, and the scattered viable glomeruli showed ischemic changes and segmental scarring. Mild glomerulitis, congestion, and fibrin microthrombi were identified in the viable glomeruli (Figure 5). Marked chronic allograft vasculopathic changes in the form of severe fibrointimal thickening and presence of intimal and subintimal foamy aggregates were readily seen in the interstitial blood vessels (Figure 6). Peritubular capillaritis was noted, of which most of the inflammatory cells were of mononuclear type. No viral cytopathic effect was identified. Immunohistochemical staining for C4d (dilution 1 : 40, polyclonal Abcam, Cambridge, UK) showed diffuse linear staining in peritubular capillaries in the renal cortex and medulla, indicating a concurrent active antibody-mediated rejection (Figure 7). Immunostaining for polyoma virus (dilution 1 : 200, clone Pab416, Abcam) was negative.

 Immunologically, anti-HLA (PRA) antibody profile revealed the following specificities: Class I-A1, 24, 80, 34, 32, 68, 36, 33, 11, and 23 (A1 and A10 CREGs) and B51. Profiling for Class II PRA was negative. The donor HLA typing was not performed, since the transplantation was performed abroad.

## 3. Discussion

Spontaneous renal allograft rupture is one of the most serious complications of kidney transplantation [5, 9–11], which typically occurs within three weeks after transplantation [1]. The prevalence of renal allograft rupture (RAR) varies from 0.3% to 3% [5]. The pathogenesis of RAR is still not fully understood. Major precipitating factors include acute rejection, ischemic acute tubular epithelial cell damage, renal vein thrombosis, mechanically damaged hilar lymphatics, and ureteral obstruction [1, 5–7]. Rarely RAR can be triggered by graft biopsy [11]. Our case showed the typical gross and histological findings of the graft rupture.

One possible mechanism put forward for RAR is that cortical and capsular ischemia results from interstitial oedema and cellular inflammatory cell infiltration causes capsular tension and rupture. The main clinical manifestations of RAR are sudden onset of abdominal pain, graft bulging and tenderness, and haemorrhagic shock [5]. Recipients of non-heart-beating donor kidneys are at a greater risk of developing graft rupture, and this has been attributed to the higher rate of acute tubular necrosis in this type of grafts. Other risk factors are high peak PRA and younger recipient, probably due to more vigorous immunological responsiveness [6]. On the other hand, the use of ATG was found to be associated with a lower incidence of RAR, presumably due to reduced frequency and severity of rejection [6]. The diagnosis is usually made based on the typical clinical picture and conventional CT scan. In our case, the CT scan findings were helpful in the diagnosis and showed intrarenal haemorrhage and retroperitoneal bleed. There is new evidence showing superiority of multidetector computed tomography (MDCT) in the diagnosis of RAR [12]. The most common course of management in RAR is urgent graft nephrectomy; however, recent reports suggest that ruptured kidney grafts are potentially salvageable by conservative surgical repair of the rupture, with good success rates [2, 5–7, 13, 14]. This approach was not considered in our case as the patient was *clinically* labelled with advanced graft failure.

Our case has the longest time interval between the time of transplant and the graft rupture in the literature. Nevertheless, the underlying cause of rupture was still severe combined acute cellular and antibody-mediated rejection, resulted from cessation of immunosuppression. Case with the second longest interval (21 months) was also a case of RAR secondary to acute rejection that followed discontinuation of immunosuppression following chronic allograft failure [15].

In our case, the patient refused the renal allograft biopsy twice, leading to the clinical assumption of allograft failure without tissue diagnosis. Although severe chronic interstitial fibrosis and tubular atrophy (IF/TA) and chronic allograft arteriopathy were evident in the graft, severe acute cellular as well as antibody-mediated allograft rejection was severely affecting the graft. Our case indicates that RAR remains a possibility five years after transplantation, particularly when immunosuppressive agents are discontinued. This raises a question of what is the best approach for immunosuppressive drug withdrawal following graft failure; should it be abrupt or gradual? The answer for this is not clear. Abrupt cessation of immunosuppression might lead to graft rejection and subsequent RAR, as in our case. On the other hand, higher morbidity and mortality have been reported in patients continued on immunosuppressive therapy after graft failure and commencement of dialysis [16]. In our experience, the presence of advanced graft failure in the form of marked interstitial fibrosis and tubular atrophy is associated with safe abrupt cessation of immunosuppressive therapy, emphasizing the value of kidney graft biopsy before discontinuation of immunosuppression medications. Messa et al. [17] described a strategy to withdraw the immunosuppressive agents in patients with graft failure whereby, the antiproliferative agents are stopped immediately to reduce the risk of infection on patients starting the dialysis, and the calcineurin inhibitors are withdrawn over 1–3 weeks if the graft failure is slow and prolonged, and 4–8 weeks if the graft failure follows a short course of time or is immunologically mediated.

Another important point to be emphasized is the role of renal allograft biopsy before the presumption of graft failure and withdrawal of the immunosuppressive agents. Our patient might started rejecting the graft long time before his initial clinical presentation 48 months after transplantation. A graft biopsy at that time would help identifying the immunological insult to the graft, dictating appropriate management, and preventing a subsequent graft rupture. Refusing graft biopsy is not uncommon in some regions and cultures of the world, and the patients should be educated about the reasons and benefits of the graft biopsy, in attempt to deliver best clinical practice.

Although the donor HLA typing was not performed in our patient, the presence of PRA Class I specificities for CREGs A1 and A10 and B51 suggests that the antibody-mediated component in the rejection process is mediated by donor-specific antibodies. Most intriguing is what may initiate an antibody-mediated immune rejection in a sensitized patient after such a long time since transplantation. Likely possibility is that the pneumonia (as an infection) may have been the triggering stimulus in our patient.

## 4. Conclusion

Renal allograft rupture is a rare but a serious complication of kidney transplant, which usually occurs few weeks after transplantation. The most common cause is acute antibody-mediated allograft rejection. Discontinuation of the immunosuppressive therapy in patients with graft failure may be a significant risk factor for late RAR and renal allograft biopsy should be performed before withdrawal of immunosuppressive agents. Following cessation of immunosuppressive therapy, careful clinical follow-up should be practiced to detect the symptoms and signs of RAR, even long time after transplantation.
